# Supplementary material for: Clinical Performance of MAGLUMI Diagnostic Tests for the Automated Detection of Dengue Virus
Source: Viruses. 2025 Jan 14;17(1):106. doi: 10.3390/v17010106 (PMC11768712; doi:10.3390/v17010106)
Supplement: Supplementary file 1 [file viruses-17-00106-s001.zip › viruses-3374882-supplementary.pdf]

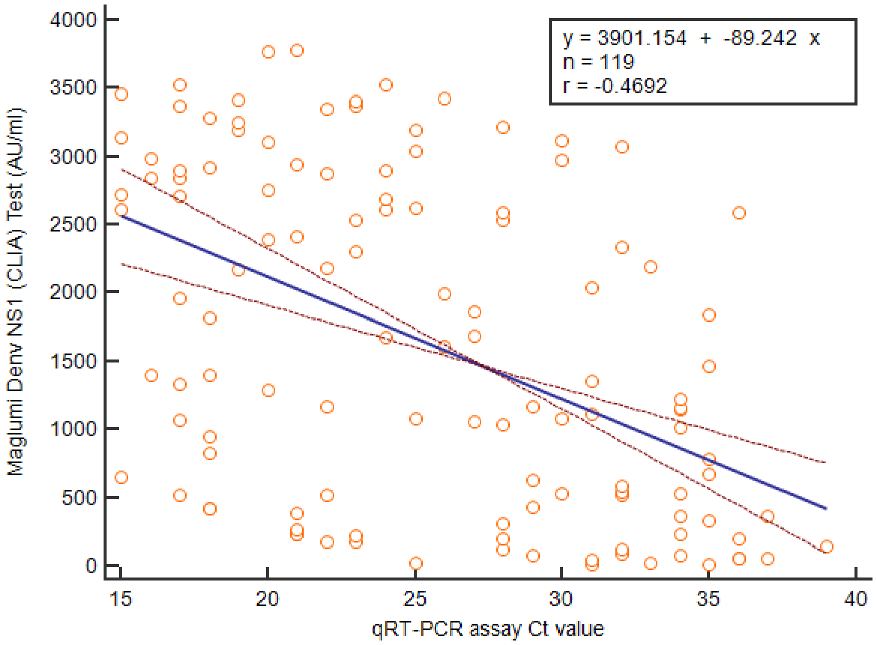

Figure S1. Correlation between the MAGLUMI Denv NS1 Test and the reference RT-PCR assay.

Table S1. Sample size, geographic origin, and clinical characteristics of 119 Dengue positive samples.

Group	Number	Constituent Ratio
Gender		
Male	91	76.47%
Famle	28	23.53%
Age		
＜18	3	2.52%
19~39	82	68.91%
40~65	31	26.05%
＞66	3	2.52%
Geographic origin		
South China	45	37.82%
Southeast/South Asia	74	62.18%
Disease course (Days)		
1-3	33	27.73%
4-6	54	45.38%
7-9	24	20.17%
10-12	8	6.72%

Table S2. 7 out of 119 cases were IgG positive and all were detected by the NS1 test.

Sample ID	qPCR Test	Dengue IgG Test	Dengue NS1 Test	Days of Fever
2	+	+	+	6
5	+	+	+	5
58	+	+	+	5
61	+	+	+	4

86	+	+	+	5
89	+	+	+	7
105	+	+	+	6
